# CHANGE IN PARENTAL DISABILITY AND INTERGENERATIONAL AMBIVALENCE: THE MEDIATING ROLE OF APPRAISAL

**DOI:** 10.1093/geroni/igac059.483

**Published:** 2022-12-20

**Authors:** Athena Koumoutzis, Kelly Cichy, Jennifer Kinney

**Affiliations:** Miami University, Oxford, Ohio, United States; Kent State University, Kent, Ohio, United States; Miami University, Oxford, Ohio, United States

## Abstract

Adult children often report intergenerational ambivalence (i.e., positive and negative sentiments toward their parents) that may be exacerbated when parents need increasing support. Evaluations or appraisal of providing support may mediate the links between stress and outcomes. Using structural equation modeling, we assessed the relationship between change in parental disability and intergenerational ambivalence through adults’ perceptions of the stress and reward of providing help to parents. Participants included 369 adults (32% Black, 68% White) who provided information on 478 parents from Waves I and II of the Family Exchanges Study. The association between change in parental disability and intergenerational ambivalence was explained through stress appraisal; greater parental disability led to higher stress appraisal which led, in turn, to greater intergenerational ambivalence. The model did not significantly differ by race. Results show that stress, rather than reward, appraisal is an essential factor in determining relationship quality as parental care needs emerge.

